# A pharmacovigilance study of Bruton’s tyrosine kinase inhibitors: a multidimensional analysis based on FAERS and VigiBase

**DOI:** 10.3389/fimmu.2025.1636657

**Published:** 2025-11-25

**Authors:** Han Qu, Yuqi Jia, Zizhen Liu, Zuan Li, Xin Zhao, Zhenghua Wu, Guorong Fan, Yuefen Lou

**Affiliations:** 1 Department of Pharmacy, Shanghai Fourth People’s Hospital, School of Medicine, Tongji University, Shanghai, China; 2 Department of Pharmacy, Peking University Third Hospital, Beijing, China; 3 Department of Pharmacy Administration and Clinical Pharmacy, School of Pharmaceutical Sciences, Peking University, Beijing, China; 4 School of Pharmacy, Shanghai Jiao Tong University, Shanghai, China; 5 Department of Clinical Pharmacy, Shanghai General Hospital, Shanghai Jiao Tong University School of Medicine, Shanghai, China; 6 Department of Clinical Pharmacy, Shanghai Jiao Tong University School of Medicine, Shanghai, China

**Keywords:** BTKis, FAERS, VigiBase, pharmacovigilance, real world study

## Abstract

**Introduction:**

The safety concerns of Bruton's tyrosine kinase inhibitors (BTKis) have garnered significant attention due to their severe adverse reactions. However, no existing studies have utilized VigiBase, the world's largest adverse event reporting system, to conduct post-marketing safety analyses of these agents.

**Methods:**

This study extracted data from VigiBase and the FDA Adverse Event Reporting System (FAERS), employing the reporting odds ratio as the primary method and information component as supplementary, to comprehensively evaluate the safety profiles of ibrutinib, acalabrutinib, and zanubrutinib, with a focus on bleeding risks when combined with anticoagulants or antiplatelet drugs.

**Results:**

The results revealed that at the system organ class level, ibrutinib had the strongest signal in cardiac disorders; acalabrutinib in blood and lymphatic system disorders; and zanubrutinib in infections and infestations and blood and lymphatic system disorders. Among the top ten standardised medical dictionary for regulatory activities queries (SMQ), the SMQs with the strongest signals were different for each BTKis, but five identical SMQs were in the top ten, namely supraventricular tachyarrhythmias, tumour lysis syndrome, haematopoietic thrombocytopenia, haemorrhage terms (excl laboratory terms), and haemorrhage laboratory terms. At the preferred term level, acalabrutinib exhibited the strongest signal for Richter’s syndrome, zanubrutinib for subcutaneous haemorrhage, while ibrutinib displayed divergent signals between databases—Bing-Neel syndrome in VigiBase and haematotympanum in FAERS. Importantly, bleeding risks varied significantly between monotherapy and combination therapy with anticoagulants/antiplatelet agents, underscoring the need for clinical vigilance regarding site-specific haemorrhage risks.

**Discussion:**

These results will provide new data to support the use of BTKis and further safety warnings. However, as a hypothesis-generating approach, it does not establish a definitive causal relationship, which will require further research and validation.

## 1 Introduction

Bruton’s tyrosine kinase (BTK) is a member of the tyrosine kinase expressed in hepatocellular carcinoma (TEC) family, and as a key kinase in the B cell receptor pathway (1, 2). BTK plays a critical role in various pivotal signaling processes *in vivo*, including B cell proliferation, trafficking, chemotaxis, and adhesion (3). Based on the crucial role of BTK, abnormalities in BTK can lead to chronic lymphocytic leukemia (CLL)/small lymphocytic lymphoma, mantle cell lymphoma (MCL), Waldenström’s macroglobulinemia, and so on (4–6). Therefore, BTK has become an essential target for treating B-cell malignancies.

In 2013, the FDA approved the marketing of ibrutinib, the first BTK inhibitor developed by Johnson Johnson and Pharmacyclics. The 2nd-generation BTK inhibitor acalabrutinib, developed by AstraZeneca, was approved for marketing by the FDA in 2017. In 2019, the Chinese pharmaceutical company BeiGene developed a Bruton’s tyrosine kinase inhibitor (BTKi) called zanubrutinib (7). In addition to this, BTKis that have been approved for marketing are tirabrutinib (8), orelabrutinib (9), and pirtobrutinib. However, due to the small number of reports in this study, they were not included to avoid unstable signals and an increase in false positive signals. Adverse drug events (ADEs) of BTKis are diverse and are concentrated in the cardiac system, dermatologic system, gastrointestinal system, infection-related, and bleeding-related ADEs (10). Currently, some studies have applied the FDA Adverse Event Reporting System (FAERS) to compare the safety of three BTKis in the occurrence of cardiac risks (11), urinary tract infection (12), and bleeding ADEs (13). A study also analyzed the safety of BTKis alone and in combination with other chemotherapeutic agents (14). There are no studies that have analyzed the post-marketing safety of BTKis using data from VigiBase (15), which is the world’s largest ADE reporting system. In this study, the safety of three BTKis will be analyzed at the system organ class (SOC), standardized medical dictionary for regulatory activities (MedDRA) queries (SMQ), and preferred term (PT) levels using two of the world’s largest spontaneous reporting systems (SRSs), FAERS and VigiBase. Hemorrhagic events are a major safety concern in targeted cancer therapy, as they may lead to treatment interruption, disease progression, or death (16). Patients receiving BTKis often require concomitant antithrombotic therapy for atrial fibrillation (AF), ischemic heart disease, or venous thromboembolism—conditions that may themselves be precipitated or exacerbated by BTK inhibition (17). The concomitant use of BTKis with anticoagulant or antiplatelet agents produces additive impairment of hemostasis, markedly increasing the risk of severe bleeding complications (18, 19). So we also analyzed the risk of bleeding when BTKis are used alone or in combination with anticoagulants/antiplatelets, which is expected to provide more data support and further reference for the safe and rational use of BTKis in clinical practice.

## 2 Methods

### 2.1 Data sources and procedures

We profiled the ADEs of BTKis by extracting data from FAERS (from the inception to Q2, 2024) and VigiBase (from the inception to May 6, 2024). The FAERS is a publicly accessible database that collects extensive reports of ADEs observed in clinical practice, providing a valuable resource for exploring potential links between drug use and ADEs (16). We used the open tool OpenVigil 2.1 and R 4.4.2 to mine and clean pharmacovigilance data from FAERS. VigiBase includes over 35 million individual case safety reports (ICSRs) of suspected ADEs (as of May 2024) submitted by national pharmacovigilance centers since 1968. We performed a disproportionality analysis of BTKis in the two international SRSs to improve the reliability of the results. We included ICSRs designated as “primary/secondary suspected” or “interacting” but excluded those characterized as “concomitant”. All ADEs were coded using MedDRA version 26.1. The narrow version of the SMQ was used because it provides better predictability while retaining comparable sensitivity to the broad version (20).

### 2.2 Statistical analysis

First, the disproportionality analysis was calculated based on **Supplementary Table S1**. In this table, N11 represents the number of cases reporting the target ADE while using the target drug; N10 represents the number of cases reporting non-target ADEs while using the target drug; N01 represents the number of cases reporting the target ADEs while using other drugs; and N00 represents the number of cases reporting non-target ADEs while using other drugs. These values were used for signal detection analysis to evaluate the potential association between the target drug and the target ADEs. We employed the reporting odds ratio (ROR) to indicate the presence of risk signals. Because of the small number of co-medication reports, we exceptionally used the information component (IC) method, an assay suitable for a small sample size, for signal mining (21). Equations (1) and (2) present the formulas for calculating the ROR and its 95% confidence interval (CI), whereas equations (3), (4), (5), and (6) describe the calculation of the IC and its 95% CI. (22).

(1) $$\begin{array}{c} ROR=\frac{N_{11}/N_{10}}{N_{01}/N_{00}} \end{array}$$

(2) $$\begin{array}{c} ROR(95\%CI)=e^{lnROR\pm 1.96\sqrt{\frac{1}{N_{11}}+\frac{1}{N_{10}}+\frac{1}{N_{01}}+\frac{1}{N_{00}}}} \end{array}$$

(3) $$\begin{array}{c} \gamma =\frac{{\left({\text{N}}_{++}+2\right)}^{2}}{\left({\text{N}}_{1+}+1)({\text{N}}_{+1}+1\right)} \end{array}$$

(4) $$\begin{array}{c} E(\text{IC})={\text{log}}_{2}\frac{({\text{N}}_{11}+1){\left({\text{N}}_{++}+1\right)}^{2}}{\left({\text{N}}_{++}+\gamma )({\text{N}}_{1+}+1)({\text{N}}_{+1}+1\right)} \end{array}$$

(5) $$\text{Var}(\text{IC})\approx {\left(\frac{1}{\log2}\right)}^{2}[\frac{N_{++}-N_{11}+\gamma -1}{\left(N_{11}+1)(N_{++}+\gamma +1\right)}+\frac{N_{++}-N_{1+}+1}{\left(N_{1+}+1)(N_{++}+3\right)}+\frac{N_{++}-N_{+1}+1}{\left(N_{+1}+1)(N_{++}+3\right)}]$$

(6) $$\begin{array}{c} \mathrm{IIC(95\%CI})\approx E(IC)\pm 2\sqrt{Var(IC)} \end{array}$$

A signal with disproportionate reporting is defined as having an ROR_025_ > 1 and at least 3 cases, or an IC_025_ > 0 (22, 23). Signal prioritization employed ROR_025_ values derived from VigiBase, the higher-reporting-volume database, to ensure analytical robustness. Patient demographic distributions and disproportionality metrics were processed through Microsoft Office Excel 2021 and Power BI.

## 3 Results

### 3.1 Patient characteristics

**Figure 1:**
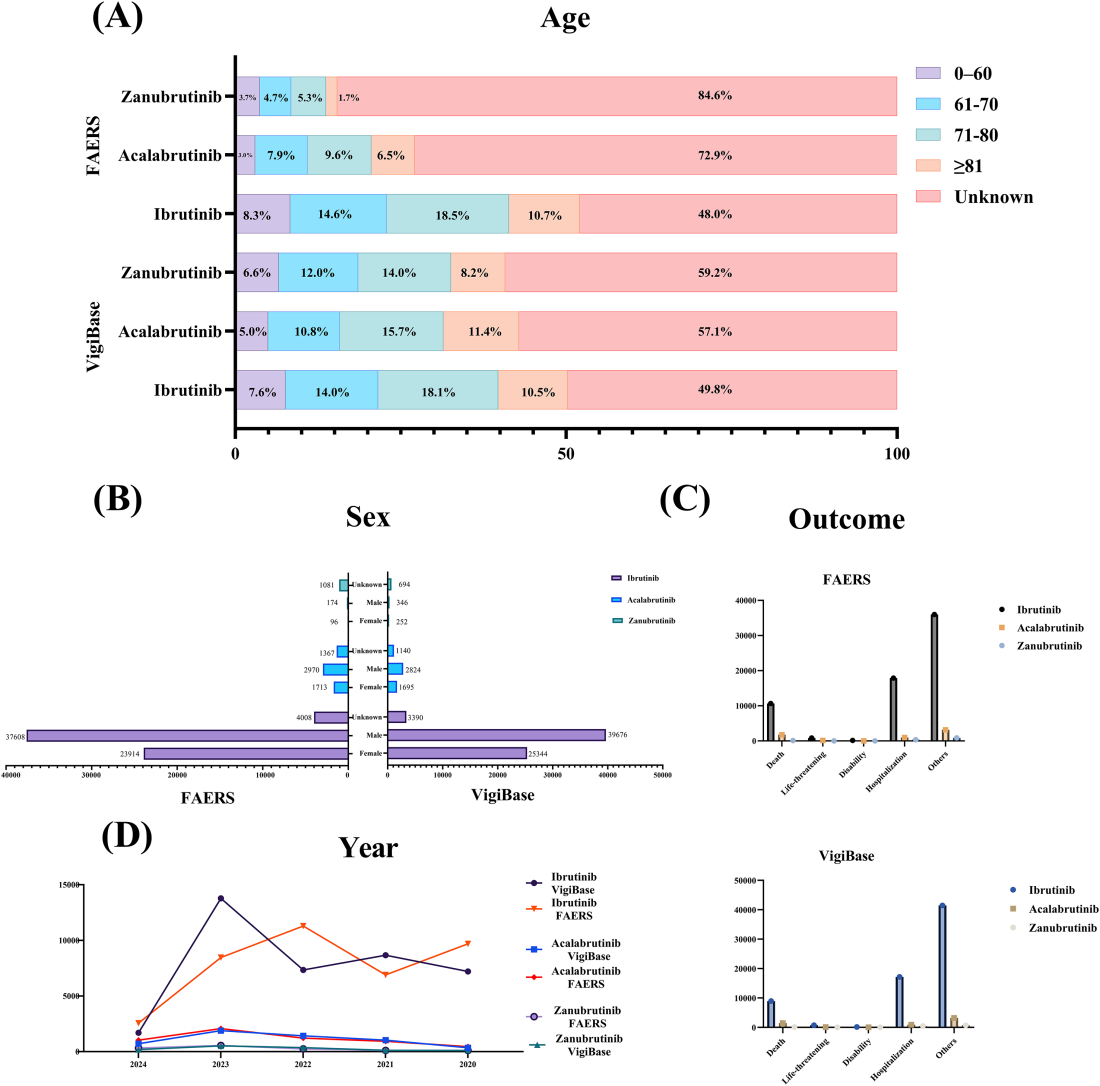
Patient characteristics of BTKis-related ADEs reports in VigiBase and FAERS. **(A)** Stacked barcharts for age groups (0-60, 61-70,71-80, 81 and above, unknown) reporting on FAERS and VigiBase databases for Zanubrutinib, Acalabrutinib, and lbrutinib. **(B)** A bar graph displaying sex distribution of reports in FAERS and VigiBase for the three drugs. **(C)** Outcome bar charts for FAERS and VigiBase, categorized by outcomes such as death, hospitalization, and others. **(D)** Line graphs tracking annual reports from 2020 to 2024 on drugs across both databases.

As shown in **Figure 1**; **Supplementary Table S2**, ibrutinib, acalabrutinib, and zanubrutinib collected 68410, 5659, and 1292 reports in the VigiBase; and 65530, 6050, and 1351 reports in the FAERS, respectively. When we do not consider unknown data, the proportion of reports was greater in males than in females and was highest in the 71–80 age group. In addition, the proportion of deaths was highest for acalabrutinib and lowest for zanubrutinib.

### 3.2 Signal strength differences of BTKis at the SOC level

**Figure 2:**
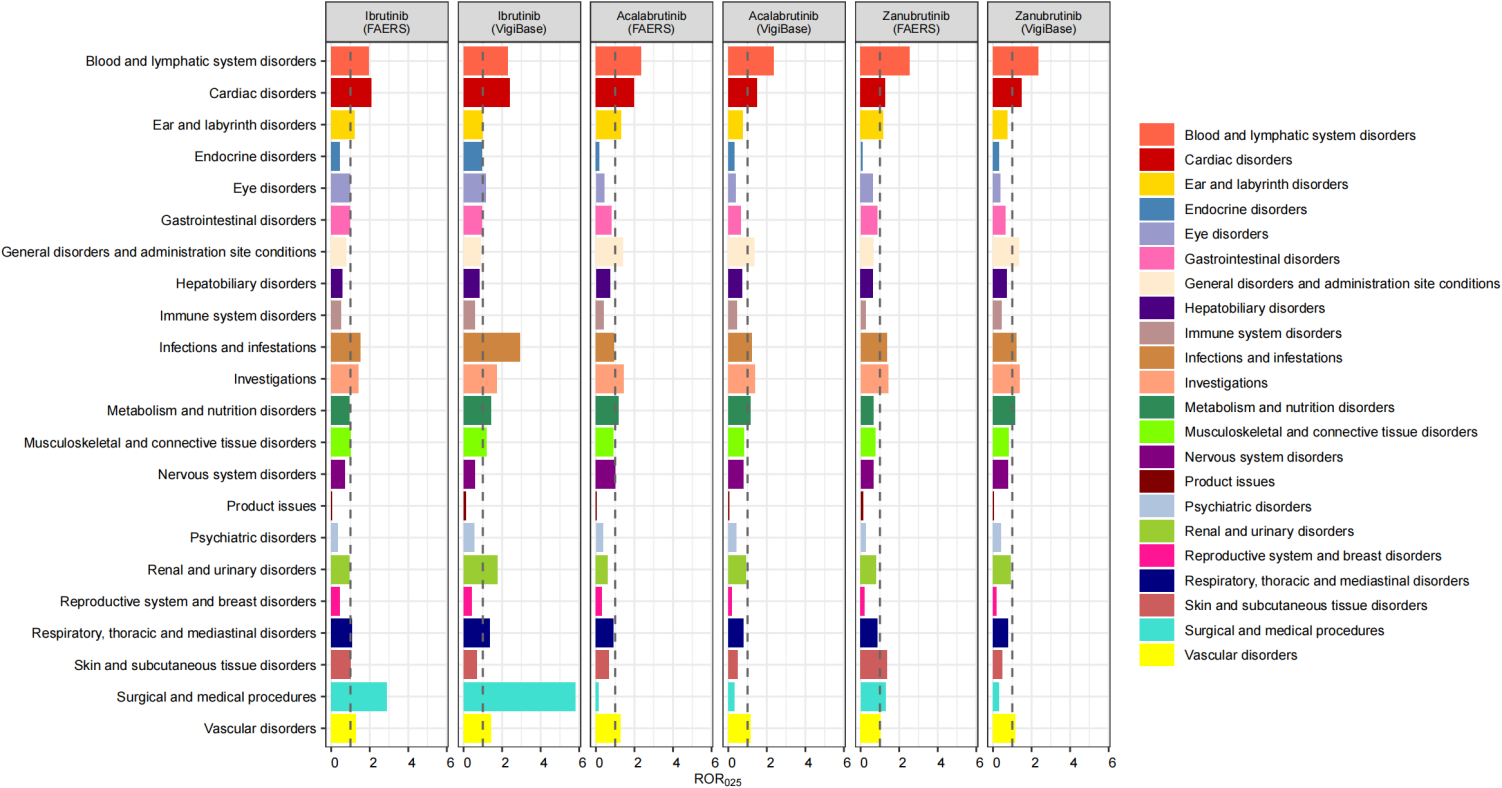
ROR_025_ for each BTKi at the SOC level in FAERS and VigiBase.

For the SOCs of the three BTKis, we excluded SOCs unrelated to the mechanism of action of the drugs, including injury, poisoning and procedural complications, congenital, familial and genetic disorders, social circumstances, neoplasms benign, malignant and unspecified, and pregnancy, puerperium and perinatal conditions. In both SRSs, significant signals were found for each BTKi in blood and lymphatic system disorders, vascular disorders, investigations and cardiac disorders. However, there were slight differences in the intensity of the signals between the drugs. Specifically, zanubrutinib had a lower signal intensity in vascular disorders but the strongest signal in blood and lymphatic system disorders than the other two drugs. The signal for the cardiac disorders was strongest in ibrutinib. In addition, only acalabrutinib suggested risk signals regarding metabolism and nutrition disorders in both SRSs. Significant signals for respiratory, thoracic and mediastinal disorders, and musculoskeletal and connective tissue disorders were only suggested for ibrutinib in both SRSs. Further, the signals for infections and infestations were significant in all 3 BTKis (no significant signals for acalabrutinib in FAERS). We also found that all 3 BTKis suggested signals in ear and labyrinth disorders in FAERS but not in VigiBase. Details of the signal strength distribution across individual drugs are presented in **Figure 2**.

### 3.3 Signal strength of BTKis at the SMQ level

The VigiBase database does not provide ADE signal data for all SMQs; therefore, the SMQ-level ADE signal data is solely derived from FAERS. We collected 65 positive signals for ibrutinib, 36 for acalabrutinib, and 40 for zanubrutinib in the SMQ hierarchy. After excluding tumor-related SMQs, the top ten SMQs ranked based on ROR_025_ are shown in **Table 1**. Among them, there are 5 identical SMQ signals that are all ranked in the top ten of the three BTKis. Ibrutinib had the highest risk of supraventricular tachyarrhythmias; acalabrutinib had the highest risk of tumor lysis syndrome; zanubrutinib had the highest risk in the other 3 SMQs.

**Table 1:** Top ten SMQs for each BTKi in FAERS.

Ibrutinib		Acalabrutinib		Zanubrutinib	
SMQ	n/ROR_025_	SMQ	n/ROR_025_	SMQ	n/ROR_025_
Supraventricular tachyarrhythmias	2182/9.86	Tumor lysis syndrome	63/20.26	Haemorrhage laboratory terms	12/7.23
Tumor lysis syndrome	144/6.37	Supraventricular tachyarrhythmias	145/4.51	Agranulocytosis	97/6.56
Lens disorders	557/4.28	Haemorrhage laboratory terms	21/3.33	Tumor lysis syndrome	7/5.18
Hematopoietic thrombocytopenia	1330/3.23	Hematopoietic erythropenia	37/3.01	Hematopoietic thrombocytopenia	68/4.40
Haemorrhage terms (excl laboratory terms)	6456/3.02	Hematopoietic thrombocytopenia	137/2.38	Supraventricular tachyarrhythmias	40/4.33
Haemorrhage laboratory terms	127/2.84	Arrhythmia related investigations signs and symptoms	4/2.33	Haemorrhage terms (excl laboratory terms)	250/4.30
Hemolytic disorders	164/2.80	Hemolytic disorders	19/2.07	Opportunistic infections	47/3.57
Tachyarrhythmia terms nonspecific	95/2.74	Cardiomyopathy	38/1.95	Hematopoietic leukopenia	93/3.04
Infective pneumonia	2009/2.73	Haemorrhage terms (excl laboratory terms)	492/1.82	Sepsis	36/2.85
Arrhythmia related investigations signs and symptoms	27/2.72	Agranulocytosis	97/1.64	Infective pneumonia	72/2.59

### 3.4 Signal strength differences of BTKis at the PT level

**Figure 3:**
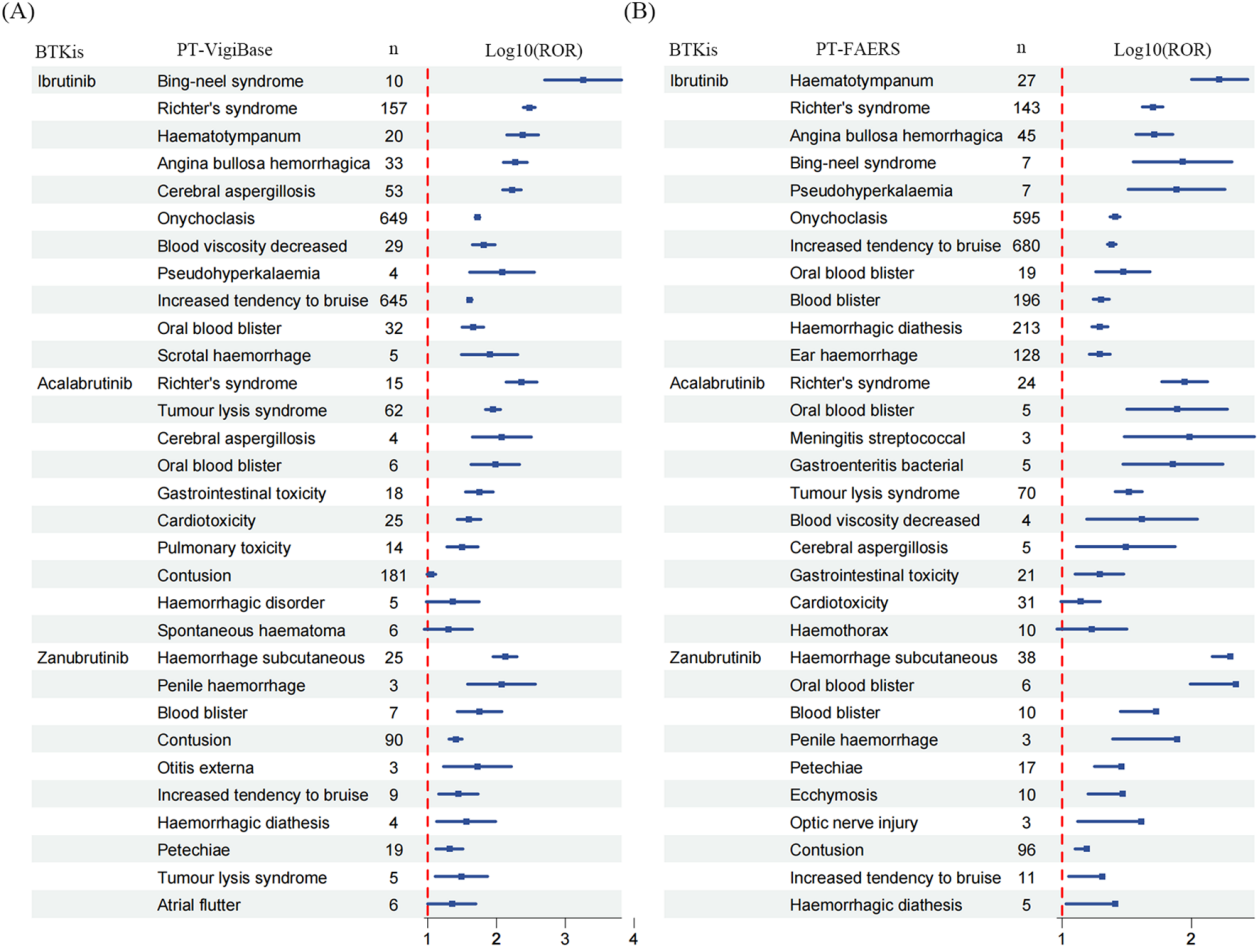
The top ten PTs for BTKis ranked by ROR_025_ in **(A)** VigiBase and **(B)** FAERS.

We first listed the top ten ADEs regarding signal strength for the three BTKis (**Figure 3**). Although the absolute signal values varied between the two SRSs, bleeding-related ADEs consistently showed prominent signal strengths across all three drugs, except for hematologic malignancy-related complications such as Richter’s syndrome and Bing-Neel syndrome. Notably, zanubrutinib was associated with an especially strong signal for subcutaneous hemorrhage. More than half of the top ten PTs ranked by signal strength for zanubrutinib were bleeding-related, underscoring the need to carefully monitor bleeding risks. Other bleeding-related signals for ibrutinib and zanubrutinib were comparable in strength. In contrast, bleeding-related ADEs accounted for only 20% to 40% of the top ten PTs for acalabrutinib (in FAERS and VigiBase reports, respectively). However, acalabrutinib showed particularly prominent signals for infection-related ADEs. According to FAERS reports, meningitis streptococcal, bacterial gastroenteritis, and cerebral aspergillosis were among the top ten reported ADEs for acalabrutinib. Despite this, the ROR_025_ for cerebral aspergillosis was still lower than that associated with ibrutinib, which continued to exhibit stronger signals for infection-related events overall. Importantly, among the three BTKis, acalabrutinib exhibited the most prominent signals for cardiotoxicity and gastrointestinal toxicity, which warrants close monitoring and further investigation into its long-term safety profile.

We also analyzed the ADEs in cardiac disorders. Ibrutinib, acalabrutinib, and zanubrutinib showed 44, 13, and 3 overlapping cardiac-related positive signals in two SRSs, respectively. All three BTKis detected signals for acute myocardial infarction, AF, atrial flutter, cardiac failure, pericardial effusion, and ventricular tachycardia in at least one SRS. Among these, ibrutinib consistently demonstrated the strongest signals for all ADEs except acute myocardial infarction. Both ibrutinib and acalabrutinib showed AF as the strongest signal in the two SRSs. Zanubrutinib had pericardial effusion and atrial flutter as the strongest signals in FAERS and VigiBase, respectively. Acalabrutinib and zanubrutinib exhibited comparable signal strengths for AF, while acalabrutinib displayed the weakest signals for atrial flutter, pericardial effusion, and cardiac failure, suggesting a lower likelihood of these specific ADEs with acalabrutinib use. However, acalabrutinib’s positive signals for acute myocardial infarction, angina pectoris, and acute coronary syndrome indicate that special attention to the prevention of coronary atherosclerotic heart disease may be required during its clinical application. All positive signals related to the cardiac disorders in both SRSs are shown in **Supplementary Material 2**.

### 3.5 Comparative analysis of hemorrhagic risk profiles

**Figure 4:**
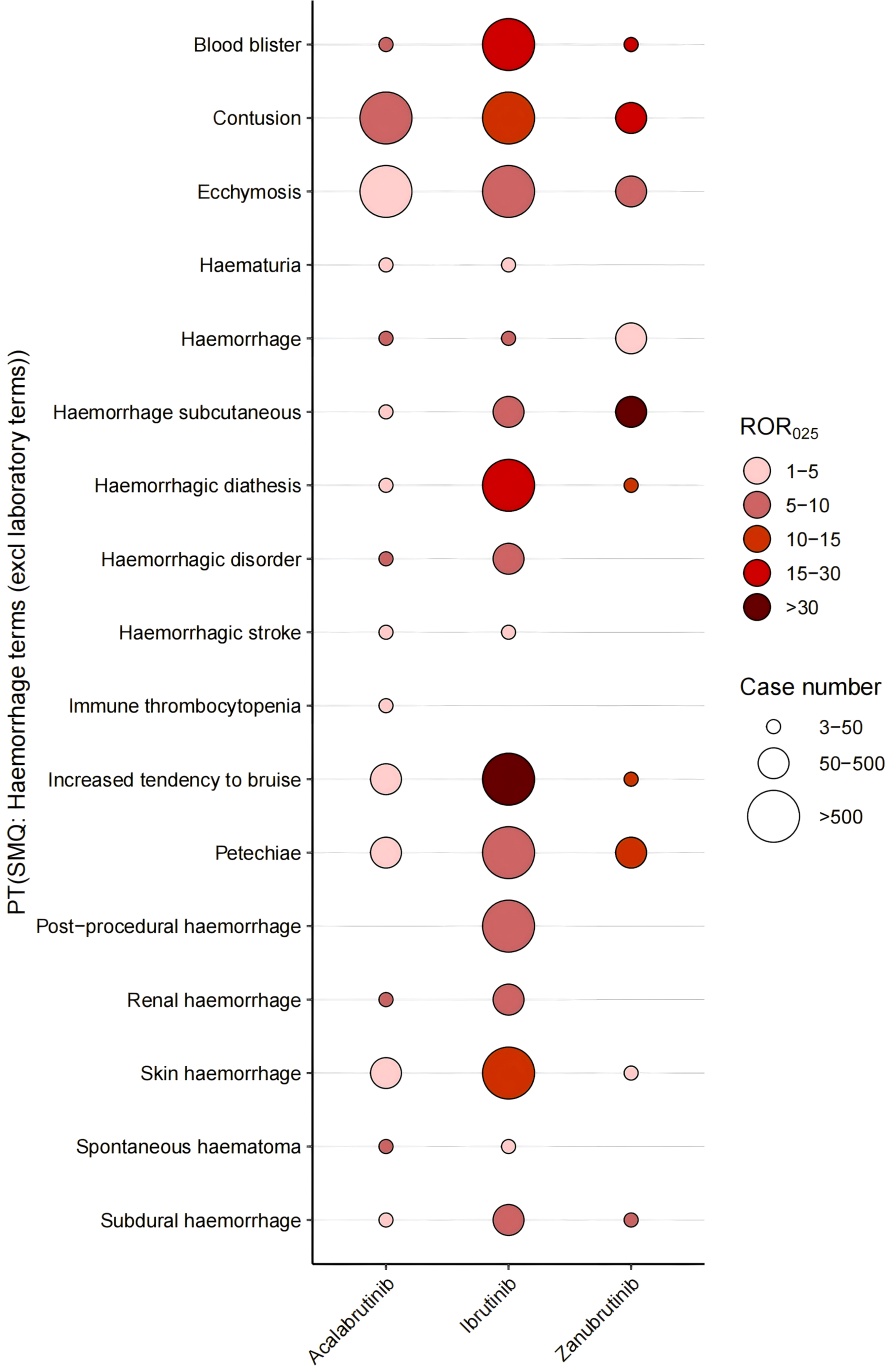
Positive signals of BTKis under haemorrhage terms (excl laboratory terms) (SMQ).

We first analyzed the hemorrhage-associated PT risk signals for each BTKi in VigiBase. As a whole, zanubrutinib had the strongest signal, and acalabrutinib had the weakest signal. Ibrutinib demonstrated the highest number of hemorrhage-related signals, exhibiting the strongest disproportionality for the majority of bleeding-associated PTs. However, acalabrutinib showed superior signal strength for hemorrhagic disorder and spontaneous hematoma, while zanubrutinib displayed heightened signals for blood blister, contusion, petechiae, hemorrhage subcutaneous, procedural hemorrhage, subdural hemorrhage, hematuria and hemorrhagic stroke (**Figure 4**). The detailed results are shown in **Supplementary Table S3**.

Second, we mined the risk signals of BTKis in combination with aspirin, clopidogrel, apixaban, and rivaroxaban, and the results are shown in **Table 2**. Due to the very limited reports of zanubrutinib and acalabrutinib co-administered with anticoagulant/antiplatelet agents, we additionally employed the more sensitive IC method to enhance early signal detection. For methodological consistency, we applied the same approach to ibrutinib, despite its comparatively larger number of reports. For zanubrutinib, a significant signal was seen in combination with apixaban when using the IC method only. Currently, the signal strength appears slightly lower than that of monotherapy. However, given that only 6 cases have been reported for the combination therapy, ongoing monitoring of case numbers and signal intensity is required. In contrast, combining acalabrutinib with clopidogrel did not show signal enhancement of bleeding risk. Notably, in the VigiBase, signal intensity for acalabrutinib in combination with apixaban or rivaroxaban exceeded its monotherapy baseline level, suggesting that specific drug combinations may influence risk performance. Ibrutinib in combination with all anticoagulant/antiplatelet agents tested showed significantly higher bleeding risk signal intensity than its monotherapy group. This finding suggests that the combination therapy of ibrutinib with anticoagulant/antiplatelet agents may increase ADEs of bleeding, resulting in a cumulatively higher risk of bleeding. In conclusion, the four anticoagulant/antiplatelet agents exhibited the strongest bleeding ADEs signal in combination with ibrutinib.

**Table 2:** Haemorrhage risk of BTKis combined with other anticoagulants/antiplatelet agents in VigiBase.

Treatment regimen	Ibrutinib			Acalabrutinib			Zanubrutinib		
	n	ROR_025_	IC_025_	n	ROR_025_	IC_025_	n	ROR_025_	IC_025_
single drug	10141	3.62	1.69	578	2.21	1.06	238	4.15	1.83
with aspirin	205	93.44	3.99	4	/	0.99	/	/	/
with clopidogrel	69	75.25	3.74	2	/	/	/	/	/
with apixaban	203	76.54	3.94	9	12.77	1.98	6	/	1.70
with rivaroxaban	204	82.23	3.96	7	18.23	1.86	/	/	/

" / " indicates no positive signal detected.

## 4 Discussion

This study is a comprehensive analysis that utilizes two of the largest SRSs in the world to characterize the safety profile of BTKis in multiple ways. Analyzing the risk of ADEs for three BTKis at different levels and in combination with anticoagulant/antiplatelet agents provides more comprehensive data to support the safe use of BTKis.

Regarding cardiotoxicity, the second-generation BTKis acalabrutinib and zanubrutinib significantly reduced the risk of AF compared to ibrutinib, consistent with evidence from previous clinical trials. This divergence may stem from their enhanced kinase selectivity: ibrutinib exhibits off-target inhibition of TEC family kinases and HER2/HER4 receptors due to lower selectivity, whereas zanubrutinib and acalabrutinib selectively target BTK with minimal HER2 interaction (24, 25). HER2/HER4 play critical roles in myocardial electrical activity and contractile function: HER2/HER4 heterodimerization is a key mechanism for maintaining cardiac signaling, and ibrutinib’s irreversible inhibition of these kinases may trigger myocardial dysfunction (26, 27). Additionally, TEC kinase inhibition may reduce PI3K-AKT signaling in cardiomyocytes, indirectly promoting arrhythmias (24). In contrast, zanubrutinib and acalabrutinib exhibit higher target selectivity: both inhibit HER4 and TEC but not HER2, and differences in TEC inhibition strength do not significantly increase cardiovascular toxicity. Since HER2 is a core driver of ibrutinib-associated AF, avoiding this target by second-generation drugs results in markedly lower AF risk (26). However, it is essential to note that ibrutinib’s broad kinase inhibition leads to significant AF, while acalabrutinib, despite its higher selectivity, shows prominent cardiac toxicity signals. Zanubrutinib generally poses lower risks but requires attention to specific atrial flutter cases.

In the vascular disorders, bleeding and hypertension were the most frequently reported ADEs across all three BTKis. Both acalabrutinib and zanubrutinib were associated with a lower proportion of bleeding events compared to ibrutinib. Previous studies have demonstrated that ibrutinib significantly increases the risk of severe and overall bleeding (16, 28–31), which is largely attributed to its effects on multiple signaling pathways. For second-generation BTKis, a meta-analysis of ten studies indicated that acalabrutinib significantly increases the risk of overall bleeding, but not severe bleeding (16). Furthermore, two head-to-head phase III multicenter trials—comparing zanubrutinib vs. ibrutinib and acalabrutinib vs. ibrutinib, respectively—showed that both acalabrutinib and zanubrutinib were associated with a reduced risk of overall bleeding compared to ibrutinib, with no significant differences observed in the incidence of severe bleeding (32, 33). BTK is essential in BT/VWF-mediated aggregation-induced thromboxane A2 generation and GPIb-dependent stabilization of arterial thrombus formation *in vivo (*34). Additionally, TEC family kinases, also substrates of ibrutinib, contribute to the downstream regulation of phospholipase Cγ2 (PLCγ2) via the collagen receptor glycoprotein VI (GPVI) on human platelets. Both Tec and BTK are required for platelet activation via GPVI, and Tec has been shown to compensate for BTK deficiency in the regulation of PLCγ2 (35, 36). Moreover, ibrutinib inhibits several intracellular signaling molecules critical for platelet function, including members of the SRC family kinases and JAK3. Inhibition of SRC by BTKis has been linked to impaired platelet aggregation and hemostasis, contributing to increased bleeding risk (16, 37). Although both acalabrutinib and zanubrutinib are also irreversible BTKis capable of selectively inhibiting BTK and Tec at low concentrations in platelets—thereby attenuating low-level GPVI-mediated platelet activation—they do not exhibit the broader off-target inhibition observed with ibrutinib. In particular, they do not significantly inhibit SRC family kinases, which play a central role in platelet signaling. Previous studies have highlighted substantial differences among the three agents’ effects on platelet function (38, 39).

In addition, our study suggests that there is a risk of bleeding when ibrutinib is combined with either anticoagulant or antiplatelet agents. Previous studies have found that about half of patients on ibrutinib develop mild to moderate platelet dysfunction, and this effect is exacerbated when combined with antiplatelet agents (40). Populations typically treated with BTKi drugs, such as those with CLL, MCL, and MZL, often have comorbidities, and there is a recognized risk of new-onset AF associated with BTKis use. Clinicians usually face challenges in treatment decision-making when combining ibrutinib with oral anticoagulants (41). Studies have suggested that acalabrutinib has superior safety characteristics concerning bleeding ADEs, particularly in patients with low sensitivity to platelet aggregation induced by ibrutinib (42), suggesting that switching BTKis in clinical practice may not reduce the risk of bleeding in all patients. Current expert consensus recommends avoiding the concomitant use of potent antiplatelet or anticoagulant agents during BTKi therapy whenever possible. If antithrombotic therapy is necessary, treatment should be initiated with a low-dose BTKi, with cautious dose escalation under close monitoring. For patients undergoing surgery or at high risk of bleeding, temporary discontinuation of BTKis for 3–7 days before and after the procedure is advised. Moreover, when combining BTKis with antithrombotic agents, an individualized risk–benefit assessment should be conducted, balancing thrombotic protection against bleeding risk and considering patient-specific comorbidities (43).

ADEs reports associated with blood and lymphatic system disorders accounted for a large proportion of all ADEs associated with BTKis and showed a high signal intensity. Abnormalities in various tests are frequently concentrated in blood-related examinations. In the usage of all three drugs, signals for abnormal platelet, white blood cell (WBC), and neutrophil counts were reported. In two head-to-head studies, hematologic toxicity had a high incidence among all ADEs. However, acalabrutinib and zanubrutinib did not demonstrate a significant advantage over the first-generation BTKis, ibrutinib, in reducing hematologic toxicity (32, 33). Other real-world studies have also monitored new hematological ADEs, such as splenomegaly and lymphadenopathy. Splenomegaly may affect blood filtration and immune function, while lymphadenopathy suggests potential local or systemic immune response abnormalities (44). It is essential to closely monitor patients’ immune and coagulation functions to prevent serious ADEs like bleeding or infection. In clinical practice, it is recommended to routinely conduct complete blood cell count tests before and during treatment, focusing on monitoring WBC count, platelet count, red blood cell count, and bleeding tendency assessment. If severe hematologic toxicity occurs, BTKi therapy should be paused, and treatment regimens should be adjusted based on the patient’s characteristics and risk-benefit assessment to ensure medication safety (43).

Infectious complications represent another critical category of ADEs associated with BTKi therapy. Overall, all three BTKis were associated with various types of infections. However, in terms of signal strength, the second-generation agents demonstrated a markedly reduced risk of infection-related ADEs compared to the first-generation inhibitor, ibrutinib. Across multiple SOC, including the central nervous system, lungs, skin, and systemic infections, there was a notable increase in the risk of infections caused by specific pathogens. Among these, aspergillus infections were particularly prominent. These findings suggest that patients receiving BTKi therapy, regardless of the generation of the agent, may be at elevated risk for aspergillosis. As such, preventing and managing aspergillus infections should be an important consideration in clinical practice when using these agents. Due to various disease-associated innate and adaptive immune deficiencies, patients with B-cell malignancies are particularly susceptible to infections. A meta-analysis has shown that ibrutinib use is significantly associated with an increased risk of infections in this patient population (45). A systematic review of 44 studies reported that 56% of patients receiving ibrutinib monotherapy and 52% of those receiving combination therapy experienced infection-related ADEs. Notably, approximately one-fifth of patients developed pneumonia (45, 46). The interference with BTK and interleukin-2-inducible T cell kinase signaling pathways in immune cells is considered a key mechanism underlying the increased susceptibility to infections observed with ibrutinib. Several reports have highlighted a particularly high risk of fungal infections, including invasive aspergillosis, associated with ibrutinib use—findings that are consistent with the results of our study (45). A signal was detected only for ibrutinib in a real-world research study specifically investigating the risk of urinary tract infections associated with BTKis treatment. The authors suggested that differences in marketing timelines among the three agents and the greater use of second-generation BTKis as first-line therapies, often in patients with relatively preserved immune function, may significantly influence the detection and reporting of infection-related ADE signals (12).

The impact of medications on liver and kidney function has always been an important area of concern. All three BTKis mention potential hepatotoxicity and nephrotoxicity in their product labels. In our study, we observed a small number of signals related to hepatotoxicity and renal impairment, but their signal strength was at the threshold level, suggesting weak statistical associations. Although these signals may reflect potential risks, their clinical significance should be interpreted cautiously, as they did not reach the threshold for significance. It is advisable to further verify these findings through laboratory tests, dose-dependency analyses, and long-term follow-up data. However, we also identified a few signals that may warrant attention. While the majority of renal-related ADEs were associated with bleeding and infections, such as hematuria and urinary tract infection, we observed a signal for fluid retention in both ibrutinib and zanubrutinib, with a stronger signal in ibrutinib. This suggests that attention to patients’ fluid status may be necessary when using these agents. In the liver system, we identified a signal for hepatitis B reactivation in both zanubrutinib and ibrutinib. This ADE may be related to immune suppression caused by BTKi therapy, rather than a direct hepatotoxic effect (47).

Regarding other systems, we also identified several less frequently mentioned ADEs signals. For example, ibrutinib was associated with middle ear effusion and deafness, while both acalabrutinib and ibrutinib showed signals for glaucoma and iron deficiency. Additionally, all three BTKis were linked to dehydration and appetite disorders. We also observed general disorders such as fatigue and tiredness across the three medications. In clinical practice, it is essential to conduct a multidimensional assessment based on the individual characteristics of patients, combining their baseline conditions with close dynamic monitoring. This approach will help identify ADE signals and optimize treatment strategies, ensuring continuity of care while improving patients’ quality of life.

Our study, like other pharmacovigilance studies, has several limitations. Firstly, there is an inherent bias in the data sources; SRSs may suffer from underreporting, duplicate reporting, and misreporting, and serious or new ADEs are more likely to be reported, whereas minor or common events may be underestimated. This could lead to an increased reporting rate of serious or more closely monitored ADEs over time, amplifying imbalances and thereby exaggerating differences between drugs. Second, the data sources for SRS are variable. FAERS data primarily originates from the United States, while VigiBase data is collected globally. Differences in data collection and processing methods, as well as variations in population bases, may account for discrepancies between signals observed in FAERS and VigiBase. Finally, even if the results of disproportionality analyses are significant, they are unlikely to confirm a causal relationship between drugs and ADEs and represent only a statistical association, a more definite causal relationship requires further verification.

## Data Availability

The original contributions presented in the study are included in the article/**Supplementary Material**. Further inquiries can be directed to the corresponding authors.
