# Supplementary material for: A pharmacovigilance study of Bruton’s tyrosine kinase inhibitors: a multidimensional analysis based on FAERS and VigiBase
Source: Front Immunol. 2025 Nov 25;16:1636657. doi: 10.3389/fimmu.2025.1636657 (PMC12685910; doi:10.3389/fimmu.2025.1636657)
Supplement: Supplementary file 1 [file Table1.docx]

Supplementary Material 1

Table S1 The 2$\times$2 contingency table for signal detection

|  | Target AE | Other AEs | Total |
| --- | --- | --- | --- |
| Target drug | N11 | N10 | N1+ |
| Other drugs | N01 | N00 | N0+ |
| Total | N+1 | N+0 | N++ |

Note: N: the number of reports.

Table S2 Patient characteristics of BTK inhibitor-related ADEs reports in VigiBase and FAERS

|  | VigilBase | | | FAERS | | |
| --- | --- | --- | --- | --- | --- | --- |
|  | Ibrutinib | Acalabrutinib | Zanubrutinib | Ibrutinib | Acalabrutinib | Zanubrutinib |
| Total | 68410 | 5659 | 1292 | 65530 | 6050 | 1351 |
| Sex |  |  |  |  |  |  |
| Female | 25344(37.05%) | 1695(29.95%) | 252(19.50%) | 23914(36.49%) | 1713(28.31%) | 96(7.11%) |
| Male | 39676(58.00%) | 2824(49.90%) | 346(26.78%) | 37608(57.39%) | 2970(49.09%) | 174(12.88%) |
| Unknown | 3390(4.96%) | 1140(20.14%) | 694(53.72%) | 4008(6.12%) | 1367(22.60%) | 1081(80.01%) |
| Age |  |  |  |  |  |  |
| 0–60 years | 5181(7.57%) | 281(4.97%) | 85(6.58%) | 5448(8.31%) | 182(3.01%) | 50(3.70%) |
| 61-70 years | 9593(14.02%) | 612(10.81%) | 155(12.00%) | 9542(14.56%) | 479(7.92%) | 64(4.74%) |
| 71-80 years | 12407(18.14%) | 886(15.66%) | 181(14.01%) | 12098(18.46%) | 583(9.64%) | 71(5.26%) |
| ≥81 years | 7154(10.46%) | 647(11.43%) | 106(8.20%) | 6997(10.68%) | 394(6.51%) | 23(1.70%) |
| Unknown | 34075(49.81%) | 3233(57.13%) | 765(59.21%) | 31445(47.99%) | 4412(72.93%) | 1143(84.60%) |
| Event year |  |  |  |  |  |  |
| 2024 | 1689(2.47%) | 718(12.69%) | 185(14.32%) | 2560(3.91%) | 1035(17.11%) | 323(23.91%) |
| 2023 | 13779(20.14%) | 1888(33.36%) | 514(39.78%) | 8454(12.90%) | 2069(34.20%) | 558(41.30%) |
| 2022 | 7339(10.73%) | 1418(25.06%) | 355(27.48%) | 11291(17.23%) | 1219(20.15%) | 249(18.43%) |
| 2021 | 8665(12.67%) | 1030(18.20%) | 121(9.37%) | 6897(10.52%) | 930(15.37%) | 109(8.07%) |
| 2020 | 7199(10.52%) | 353(6.24%) | 104(8.05%) | 9685(14.78%) | 452(7.47%) | 103(7.62%) |
| Outcome |  |  |  |  |  |  |
| Death | 8930(13.05%) | 1456(25.73%) | 117(9.06%) | 10644(16.24%) | 1747(28.88%) | 109(8.07%) |
| Life-threatening | 739(1.08%) | 92(1.63%) | 28(2.17%) | 848(1.29%) | 146(2.41%) | 20(1.48%) |
| Disability | 143(0.21%) | 23(0.41%) | 5(0.39%) | 143(0.22%) | 25(0.41%) | 11(0.81%) |
| Hospitalization | 17164(25.09%) | 876(15.48%) | 451(34.91%) | 17898(27.31%) | 973(16.08%) | 334(24.72%) |
| Others | 41434(60.57%) | 3212(56.76%) | 691(53.48%) | 35997(54.93%) | 3159(52.21%) | 877(64.91%) |

Table S3 Bleeding-associated signal of each BTK inhibitor alone in VigiBase

| SMQ/PT | Ibrutinib | | Acalabrutinib | | | Zanubrutinib | | |
| --- | --- | --- | --- | --- | --- | --- | --- | --- |
|  | n | ROR_025_ | | n | ROR_025_ | | n | ROR_025_ |
| SMQ: Haemorrhage terms (excl laboratory terms) |  |  | |  |  | |  |  |
| Increased tendency to bruise | 645 | 37.67 | | 10 | 3.81 | | 9 | 14.57 |
| Haemorrhagic diathesis | 183 | 28.64 | | 5 | 4.31 | | 4 | 13.63 |
| Blood blister | 182 | 25.23 | | 8 | 7.40 | | 7 | 27.09 |
| Renal haemorrhage | 29 | 7.04 | | 4 | 6.27 | | / | / |
| Contusion | 2572 | 13.10 | | 181 | 9.74 | | 90 | 20.64 |
| Haemorrhagic disorder | 24 | 6.24 | | 5 | 9.66 | | / | / |
| Skin haemorrhage | 249 | 10.32 | | 12 | 3.79 | | 4 | 3.66 |
| Petechiae | 525 | 10.00 | | 22 | 3.56 | | 19 | 13.15 |
| Post-procedural haemorrhage | 219 | 8.90 | | / | / | | 5 | 5.03 |
| Haemorrhage subcutaneous | 86 | 6.96 | | 7 | 3.99 | | 25 | 90.00 |
| Procedural haemorrhage | 59 | 6.18 | | 4 | 2.43 | | 3 | 6.86 |
| Ecchymosis | 288 | 8.09 | | 15 | 3.40 | | 7 | 5.49 |
| Subdural haematoma | 278 | 7.82 | | 11 | 2.29 | | 8 | 6.61 |
| Subdural haemorrhage | 40 | 5.17 | | 5 | 4.39 | | 3 | 8.95 |
| Haemorrhage | 1173 | 5.58 | | 105 | 5.24 | | 27 | 4.90 |
| Haematuria | 412 | 3.53 | | 17 | 1.20 | | 16 | 4.89 |
| Haemorrhagic stroke | 45 | 2.19 | | / | / | | 3 | 3.33 |
| Immune thrombocytopenia | 44 | 1.55 | | 5 | 1.19 | | / | / |
| Spontaneous haematoma | 11 | 1.68 | | 6 | 8.99 | | / | / |
